# Serious diarrhea with weight loss caused by *Capillaria philippinensis* acquired in China: a case report

**DOI:** 10.1186/1756-0500-5-554

**Published:** 2012-10-05

**Authors:** Zhigang Fan, Yongdong Huang, Shiyun Qian, Gang Lv, Yiyao Chen, Bo Yang, Saifeng Zhong, Guifen Lin, Guogang Yan

**Affiliations:** 1 School of tropical and laboratory medicine, Hainan Medical University, Haikou, China; 2 Department of Digestive Diseases, People’s Hospital of Hainan province, Haikou, China; 3 School of nursing Hainan medical college, Haikou, China

**Keywords:** Diarrhea, Intestinal capillariasis, *Capillaria philippinensis*, China

## Abstract

**Background:**

Diarrhea caused by *Capillaria philippinensis* (*C. philippinensis*) has not been reported in any areas with the exception of Taiwan province in China. We herein report the misdiagnosis and subsequent management of a patient with diarrhea caused by *C. philippinensis*.

**Case presentation:**

A 33-year-old woman from the outskirts of Danzhou city, Hainan province, China, had an 11-month history of chronic diarrhea with abdominal pain, edema, hypoalbuminemia, and severe weight loss. The patient was misdiagnosed at an outpatient clinic and one hospital. She was finally correctly diagnosed with *C. philippinensis* by stool examination. The patient was given a 30-days course of albendazole (400 mg/day) and had an uneventful and stable recovery.

**Conclusion:**

Doctors cannot lose sight of patients’ dietary histories, must query stool examination results, and need to expand their knowledge of certain nonlocal and global diseases, especially those described in new case reports. Some diagnostic examinations must be performed repeatedly. Hainan province may be the epidemic focus of *C. philippinensis*.

## Background

Parasites are rarely suspected as causes for diarrheal episodes because of their decreasing infestation rates and endemic zones. *Capillaria philippinensis* (*C. philippinensis*) is a rare food-borne nematodiasis that has appeared in more than 12 regions or countries worldwide, including the Philippines [1], Indonesia [2], Thailand [3], Lao People’s Democratic Republic [4], India [5], Iran [6], Korea [7], and Japan [8]. More than 2000 cases involving almost 200 deaths have been documented worldwide. However, no cases have been reported in China with the exception of Taiwan province [9-11]. *C*. *philippinensis* may cause diarrhea, hypoalbuminemia, and death in humans [8,12]. We herein report a case of serious diarrhea with weight loss caused by *C*. *philippinensis* from Hainan province in China.

**Table 1:** Laboratory investigations in 33-year old female patient from Danzhou in April 2010

	**Result**	**Normal value**		**Result**	**Normal value**
hemoglobin(g/L)	85	110.0 - 150.0	Total protein (g/L)	47	38.0 - 60.0
Hematokrit	0.26	0.37 - 0.44	Albumin (g/L)	14	35.0 - 55.0
mean corpuscular volume (fL)	72.2	82.0 - 95.0	Albumin/Globulin	0.4	1.0 - 2.0
Mean Corpuscular Hemoglobin(pg)	23.9	27.0 - 31.0	glutamic-oxalacetic transaminase(U/L)	80	5.0 - 40.0
Neutrophil counts(10^9^/L)	2	3.2 - 6.4	Lactic dehydrogenase (U/L)	255	110.0 - 240.0
Neutrophils percentage(%)	44.94	50.0 - 80.0	Creatine Kinase-MB (U/L)	45.7	0 - 25
eosinophilic granulocytes counts(10^9^/L)	0.06	0.1 - 0.5	Prealbumin (mg/L)	66	180 - 390
Monocyte rate (%)	11.64	3.0 - 10.0	Creatinine (umol/L)	34	44 - 115
NO2 partial pressure(mmHg)	30.9	35.0 - 45.0	Triglyceride (mmol/L)	1.92	0.33 - 1.69
Arterial partial pressure of oxygen(mmHg)	101	80.0 - 100.0	Total cholesterol (mmol/L)	2.43	3.60 - 5.17
actual bicarbomate (mmol/L)	20.6	21.4 - 27.3	Apolipoprotein A-I (g/L)	0.71	1.0 - 1.6
Total CO2 (mmol/L)	21.5	22.0 - 32.0	Apolipoprotein B-100 (g/L)	0.56	0.6 - 1.1
Bases excess (mmol/L)	−4	−3 - 3	High density lipoprotein (mmol/L)	0.78	1.17 - 2.00
Hematokrit (%)	35	37 - 44	Low density lipoprotein (mmol/L)	1.27	2.0 - 4.14
Arterial oxygin content (mL/dL)	16	17.6 - 23.6	Lipoprotein a (g/L)	0.03	0.05 - 0.3
Serum potassium (mmol/L)	2.74	3.5 - 5.3	glucose (mmol/L)	3.06	FPG3.89 - 6.11
Serum sodium (mmol/L)	127.7	136.0 - 145.0	glycosylated serum protein (umol/L)	53	122 - 236
Serum calcium (mmol/L)	1.58	2.1 - 2.9	retinol binding protein (mg/L)	10.5	25 - 70
Serum chlorinum (mmol/L)	92.1	96.0 - 108.0			

## Case presentation

A 33-year-old woman came from the outskirts of Danzhou city, Hainan province, China. In April 2010, she reported an 11-month history of recurrent diarrhea associated with colicky pain. The watery diarrhea had persisted since May 2009, occurring about three to four times daily. She also experienced weight loss of 12.5 kg. She was admitted to an outpatient clinic and one hospital in Danzhou. Since May 2009 to March 2010, the patient was given a treatment of checking diarrhea. The detailed diagnosis, treatment and lab investigation of her diarrhea were not clear. In April 2010, because her symptoms could not be relieved, she was admitted to People’s Hospital of Hainan province in Haikou, China. Clinical examination showed moderate dehydration; pallor; a soft, nontender, nondistended abdomen; and marked pitting edema of the lower limbs. The liver and spleen were not enlarged.

Serum total protein and albumin levels were low (47 and 14 g/L, respectively), and proteinuria was not detected (Table 1). A chest radiograph and abdominal ultrasound did not disclose any specific abnormalities. Gastroscopy showed hyperemia, edema, and superficial erosions in the gastric antrum.

**Figure 1:**
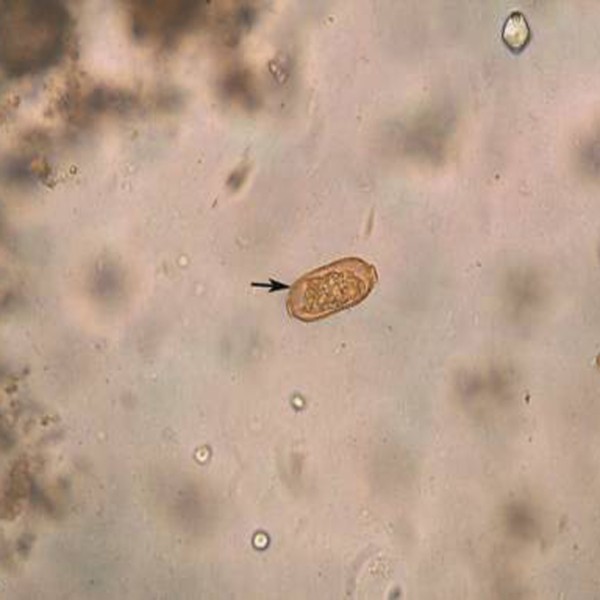
**Egg of *Capillaria philippinensis *in 33-year old female patient from Danzhou.**

In April 2010, stool examinations were quickly performed several times through direct smear method by the laboratory of the hospital, and nothing was found. In May 2010, Stool examinations were carefully performed 9 times through direct smear method and brine flotation by the Department of Parasitology of Hainan Medical College. Eggs (0–1 eggs/10 ×40 microscopic fields) that were elongated and peanut-shaped with flattened bipolar plugs and striated shells (43.7 × 14.6 μm) were observed as shown in Figure 1. The eggs were identified as those of *C*. *philippinensis*. Adult male *C*. *philippinensis* (1.6 × 0.1 mm) was also found in the stool samples. In addition, cysts of *Entamoeba coli* were found.

The patient reported eating sashimi of two raw *Misgurnus anguillicaudatus* loaches every day from March to April in 2009 to treat a 7-year history of constipation. One month later, the diarrhea associated with colicky pain emerged and persisted. She had no travel history outside the residence town in the time before falling ill. All other family members did not eat the fish, and were healthy without any diarrhea. The patient was given a 30-days course of albendazole (400 mg/day). The patient left the hospital at the fifth day of treatment. Till now, the patient had an uneventful and stable recovery through telephone follow-up visit.

## Conclusions

*C*. *philippinensis* is a helminth of the small intestine and causes severe enteropathy and, at times, death in humans. Fish and fish-eating birds are natural hosts of *C*. *philippinensis*[8]. Fish-eating birds may be infected with larvae from fish or be fed infected fish, perpetuating the parasite’s fish-bird life cycle [8]. Humans can become infected when they eat raw or insufficiently cooked infected fish [8,13]. Most female worms are oviparous, but a few female worms produce larvae, which can lead to autoinfections and hyperinfections [8]. Humans commonly eat raw fish in many areas of China, such as Guangdong province and Guangxi province. *Clonorchis sinensis* has often been reported in these areas [14], but *C*. *philippinensis* has never been reported. In addition, stool examinations showed 0 to 1 eggs for every 10 ×40 microscopic fields in the case. Therefore, the doctors should be particularly sensitized to the consumption of raw fish which should be asked during anamnesis and made aware of zoonosis which are transmitted through raw or insufficiently cooked fish. It is much less knowledge of “nonlocal and global diseases”. Some diagnostic examinations must be carefully performed several times.

This patient was infected with *C*. *philippinensis* from the *Misgurnus anguillicaudatus* loach. In China, many people eat raw loaches because these fish are thought to have very high medicinal properties. People have been infected with several parasites from loaches, such as *Clonorchis sinensis*[15], *Paragonimus westermani*[15], and *Echinostoma hortense*[16]. The *C*. *philippinensis* infection herein reported is the first to be documented in China outside of Taiwan province [9-11]. The patient ate sashimi of raw loaches because she had a 7-year history of constipation and had heard through the local elders that loaches can treat constipation. The patient had no travel history outside the residence town in the time before falling ill, so she had acquired *C*. *philippinensis* infection in Hainan. Taiwan and Hainan are geographically close to the Philippines and Thailand. Taiwan is a key rest stop on the north–south migratory routes of many fish-eating migratory birds that are thought to be the major carriers of *C*. *philippinensis*[9]. Hainan province, including Hainan Island, is located in tropical and subtropical regions. Danzhou is located in the northwest of Hainan province. There are 67 species of wintering waterbirds on Hainan island [17]. Therefore, people should change the attitude or custom of eating raw loaches for treating diseases, and Hainan province may be the natural epidemic focus of *C*. *philippinensis*.

## Consent

Written informed consent was obtained from the patient for publication of this case report and any accompanying images. A copy of the written consent is available for review by the Series Editor of this journal.

## Competing interests

The authors declare that they have no competing interests.

## Authors’ contributions

Fan ZG drafted and revised the manuscript. Huang YD, Chen YY and Yang B have been involved in patient clinical care. Fan ZG performed the the stool examination. Qian SY, Lv G, Zhong SF, Lin GF and Yan GG interpreted data and reviewed the manuscript. All authors read and approved the final manuscript.
